# No bad abortions: Graphic abortion narratives as feminist discourse

**Published:** 2023-03-30

**Authors:** Cordelia Freeman, Rishita Nandagiri

**Affiliations:** University of Exeter; LSE

## Abstract

‘Graphic medicine’ refers to the bringing together of biomedical discourses and comics in order to problematize power asymmetries within healthcare and medicine (Czerwiec et al. 2015; Green and Myers 2010). Within this, there has been increased attention on the topic of reproduction in order to challenge the medicalisation of reproduction and centre the experiences of people as they have sex, navigate contraceptions, become pregnant, give birth, and/or have abortions, among other issues. Abortion as an embodied subject in these texts has become a key symbol of feminist discourse. Whether created for political feminist reasons, as educational tools, or as emotional catharsis, abortion graphic narratives play an important and increasingly prominent role in shaping how we think about abortion. Narratives that portray abortion as a positive decision have been important for combating abortion sigma, challenging assumptions about abortion, humanising abortion seekers, and rejecting the idea that there is a simple binary of ‘good’ and ‘bad’ abortions. Here, we reflect on our own experiences as academics who have co-created graphic abortion narratives with activist groups. Cordelia worked with Fondo MARIA to co-create *Será Deaseada*, a graphic novel following three Mexican women as they seek abortions and Rishita worked with Asia Safe Abortion Partnership to co-create *Nirnay*, a series of six comics tracing women’s differing abortion trajectories in India. Through a reflective conversation we examine the power of the visual, our different processes of creating graphic narratives, and the challenges that come with it and conclude by setting out our framework for conceptualising our work as academics producing graphic narratives.

## Introduction

‘Graphic medicine’, an emerging and interdisciplinary space, is the intersection of biomedical discourses with the medium of comics (Czerwiec et al. 2015). Challenging dominant frames surrounding illness, disability, healthcare, medicine, and caregiving, graphic medicine offers a more inclusive approach (Green and Myers 2010). As a medium, comics have long explored ‘taboo’ topics; offering a rich tradition of disrupting universalist and ‘objective’ perspectives, enabling the representation of a multiplicity of voices and bodies within them, and allowing for new imaginations to flourish. As part of this space, comics are increasingly addressing reproduction, in what has recently been termed ‘graphic reproduction’. Graphic reproduction has emerged as part of a broader feminist challenge to the medicalisation of reproduction (Johnson 2018). It contends with the personal and political meanings surrounding a range of reproduction-related experiences (sex, contraception non/use, conception, pregnancy, abortion, childbirth), the emotions and contradictions that infuse them, and the experiences of navigating access to care.

Abortion as an embodied subject in these texts has become a key symbol of feminist discourse. Whether created for political feminist reasons, as educational tools, or as emotional catharsis, abortion graphic narratives play an important and increasingly prominent role in shaping how we think about abortion. Explicitly positive narratives about abortion are making space for varied experiences and have the potential to challenge abortion stigma (Ludlow 2020a). Abortion narratives disrupt normative assumptions about abortion and the experiences people face as they navigate the decision and process to have an abortion, with many abortion comics drawing on the creators’ own experiences. The humanizing potential of abortion comics forces readers to think beyond the pro-life/pro-choice binary and rejects moralizing notions of there being ‘good’ (and therefore by definition also ‘bad’) abortions (Ludlow 2019).

In this work, we first explore the depictions and narratives within the growing number of comics and graphic novels that directly deal with abortion. Heeding Belfrage, Didier & Vázquez-Quesada’s (2021) call for greater feminist reflexivity in abortion storytelling, we then draw on our own experiences as academics who have co-created graphic abortion narratives. Reflecting on our projects, *Será Deaseada*, a graphic novel following three Mexican women as they seek abortions and *Nirnay*, a series of six comics tracing women’s differing abortion trajectories in India, we examine the power of the visual, our different processes of creating graphic narratives, and the challenges that come with it. We contend with the feminist ethics of representing abortion seekers’ experiences (Dix and Kaur 2019), and the potential for illustrations to engage readers’ imaginations in new ways; destabilising static, reductive, and stigmatising frames surrounding abortion, abortion seekers, and abortion providers. We conclude the paper by setting out our framework for conceptualising our work as academics producing graphic narratives. In contrast to current frameworks which involve translating research data into visual forms, our projects involved collaborating with activist groups who fight for change within our research areas. This is a collaborative, situated, and collective approach that attempts to ground a feminist ethics of care and justice, amongst the challenges of navigating the neoliberal Global North university.

## Bridging the Graphic, Medicine, and Reproduction

Graphic medicine is broadly defined as ‘the intersection of the medium of comics and the discourse of healthcare’ (Czerwiec et al. 2015: 1). Coined by British doctor Ian Williams in 2007, it refers to comics that are used to help healthcare professionals understand their patients’ experiences and as a creative outlet for those with illnesses (Glazer 2015). These comics typically attempt to challenge power imbalances in medicine by giving voices to those who are often not heard and by disrupting the notion of the ‘objective’ case study by emphasising autobiographical experiences (Czerwiec et al. 2015). They also enable the exploration of taboo or hidden aspects of illness and healthcare (Czerwiec et al. 2015). The key difference between comics and prose, argue Green and Myers (2010) is that the visual elements of comics can provide immediate, visceral reactions.

Graphic reproduction, a nascent subfield, incorporates a wide range of reproductive experiences such as sex, contraception, conception, pregnancy, abortion, and childbirth. Graphic narratives concerning reproduction can include medical aspects, but they predominantly focus on emotions and the personal experiences of reproduction and accessing care. Jenell Johnson’s anthology *Graphic Reproduction* (2018) includes excerpts from eleven comics across a range of reproductive experiences such as abortion, miscarriage, homebirthing, and post-partum depression. Many of these comics subvert, challenge and critique medical authority, especially the medicalisation of reproduction. By reclaiming agency and voice through the privileging of these perspectives, graphic reproduction comics also function as a form of ‘talking back to’ medical authority and its structures.

### Abortion and Graphic Reproduction

The self-published comic *Abortion Eve* (1973) was a radical contribution for directly telling the stories of five women as they have abortions alongside information about anatomy and abortion techniques (McGovern and Eve 2019). *Abortion Eve* treats abortion not as a medical issue but as a societal, political, and legal issue that is directly speaking to women and this sets it apart from typical works of graphic medicine, which are usually singular and personal (McGovern and Eve 2019). Nevertheless, for McGovern and Eve (2019: 23) ‘in considering its function as a pre-runner of contemporary graphic medicine alongside its self-published nature, Abortion Eve centres itself as an extremely early graphic medical herstory’. *Abortion Eve* therefore played an important role in developing the foundations upon which other abortion comics have been able to flourish.

*Comics for Choice* (2017), for instance, an anthology of abortion comics edited by Hazel Newlevant, Whit Taylor, and ø.K. Fox, aims to ‘educate readers about many facets of the history of abortion in America the incredible diversity of reasons people choose it, and what we can do to protect this crucial right’ (1). The collection includes an impressive 41 comics that cover autobiographical abortion experiences, perspectives from clinic escorts, and from those who work in abortion healthcare. *Abortion Eve* and *Comics for Choice*, despite being published in different eras and slightly different formats, are similar in linking to broader political feminist self-help and women’s rights movements with goals of information sharing and self-education through graphic narratives. Through depictions of reproduction-related health services (e.g., pelvic examinations, anaesthesia, anaemia, contraception, abortion), the comics demystify them; contributing to the democratization of knowledge. The explicit and transparent discussion of abortion also challenges abortion stigma, and ‘at least within women’s circles, removed the frame through which society was taught to perceive it as shameful and taboo’ (McGovern and Eve 2019: 17).

Ludlow (2020b) argues that representations of abortion in pro-choice movements tend to be oversimplistic or a binary. The prolife/prochoice binary ‘organizes conversations about abortion both inside clinics and in public spaces, ultimately creating a hierarchy of abortion narratives according to which some abortions are perceived to be appropriate, more defensible, and more respectable, than others’ (Ludlow 2019: 185). In particular, she critiques pro-choice movements for not offering images that effectively counter anti-abortion images of foetal materiality; instead making the aborted foetal body an absent presence.

Ludlow (2020a: 119) also argues that narratives that attempt to ‘normalise’ abortion can be apologetic or frame abortion as a necessary tragedy where ‘“normalization” participates in a poetics of concession’. In her analysis of the abortion comic *Not Funny Ha-Ha*, Ludlow (2020a: 118) argues that the constant repetition that having an abortion is ‘fine’, instead ‘serves to draw attention to just how un-fine it might be’. She also laments that the two characters in the graphic novel aren’t shown as happy or relieved after their procedures. This echoes Erica Millar’s (2017) argument that abortion is persistently framed as ‘unhappy’ and ‘difficult’, even by pro-abortion proponents, which negates the possibility of a ‘happy abortion’. Thomsen (2013) calls for a new and collective reimagining of abortion depictions, one that moves away from negative framings to one that views abortion as ‘something *worthy of celebration’* (155). Similarly, in an analysis of sex education comics, Faris (2019) suggests that explicitly feminist and queer positionalities can teach sex and sexuality as a techné– ‘civic practices that are generative of new ways of relating with others and being in the world’ (86). This techné can thus re-orient understandings of body and society, challenging and critiquing norms.

### Abortion storytelling: counter-narratives, resistances, and reproductive justice

Beyond graphic narratives of abortion, abortion storytelling more broadly has been celebrated for its potential to reduce stigma, bring about legislative change, give voice to those silenced, and combat isolation (Belfrage, Didier, and Vázquez-Quesada 2021). In their work on ‘counter narratives’ of abortion, Baird and Millar (2019) argue that it is important to understand what feminist and pro-choice narratives of abortion are actually doing. They use the term ‘unapologetic’ representations of abortion and argue that while they have been important, they can reify the neoliberal autonomous subject; leading to simplified abortion narratives that smoothen out differences.

While much of abortion activism and storytelling has been framed within the rhetoric of ‘choice’ or ‘healthcare’ (Price 2020), Reproductive Justice (RJ) offers a more situated and contextual understanding of abortion and reproduction. Conceptualised by Black feminists in USA, RJ critically examines the structural mechanisms and contexts (e.g., race, gender, sexualities, immigration status) that shape peoples’ reproductive lives (Ross 2017). RJ champions three tenets: (i) the right *to* have a child under conditions of one’s own choosing; (ii) the right *not* to have children and (iii) the right *to parent* children & raise families in safe and healthy environments (Ross and Solinger 2017). Locating abortion within these broader contexts and structures, and accounting for matrixes of oppressions, RJ accounts for a feminist articulation of differences within abortion narratives and storytelling.

‘…storytelling is an act of subversion and resistance. Stories help us understand how others think and make decisions. They help us understand how our human rights - and the human rights of others - are protected or violated. Storytelling is a core aspect of reproductive justice practice because attending to someone else's story invites us to shift the lens - that is, to imagine the life of another person and to reexamine our own realities and reimagine our own possibilities.’

(Ross and Solinger 2017: 59)

Comics can be immensely powerful in representing the private, secret, and invisible/invisibilised experiences and thoughts (Ludlow 2019), but feminist activists and collectives must pay attention to contexts, how abortion storytelling is utilised and to which ends. Personal stories must be carefully curated to avoid silencing, cooptation, or erasure of women’s experiences (Belfrage, Didier & Vázquez-Quesada 2021). In contexts where abortion remains restricted, criminalised, and/or stigmatised, taking a reproductive justice approach to storytelling can draw attention to structural inequalities and injustices (Belfrage, Didier & Vázquez-Quesada 2021: 13).

Many graphic abortion and graphic medicine narratives tend to focus on the USA (Belfrage, Didier & Vázquez-Quesada 2021; Williams 2012), but there is a growing body of literature from Global South and non-Anglophone contexts. These include *AbortoS en Plural* (2020) by Las Comadres in Ecuador, *Todas Nosotras* (2020) by Elizabeth Casillas and Higinia Garay about four Salvadorian women, *Papaya Parade* (2020); a series of short narratives about abortion in India, and Safer Abortion’, a series of videos and comics coordinated by PositiveNegatives and based on adolescents’ abortion experiences in Malawi, Zambia, and Ethiopia (2020). These graphic narratives reflect a range of contexts (restrictive and more liberal legal contexts), individual circumstances (e.g., age, marital status), and abortion methods (self-managed, in formal health clinics). Like *Abortion Eve* and *Comics for Choice*, they contribute to information sharing and education, expanding representations of abortion and offering a counter narrative. These representations, as a techné of abortion, challenge abortion stigma and norms; re-orienting understandings of abortion (Faris 2019).

In the following sections, we respond to Belfrage, Didier & Vázquez-Quesada’s (2021) call for a reflexive and feminist approach to abortion storytelling, locating our graphic abortion narratives from Mexico and India within reproductive justice.

## Creating Collaborative Abortion Graphics

### Origin stories

Feminist reflexivity, and writing about it, are situated acts (Nencel 2013), enabling an analysis of the researcher‘s (and their study) relationships(s) with power (Kobayashi 2003; Sultana 2007). Braidotti (2009: 243) links such self-scrutiny to a critical self-narrative, where ‘ ‘embodied’ accounts illuminate and transform our knowledge of ourselves and of the world’. Thus, we briefly introduce our projects and contextualise our approaches before offering conversational responses to a series of reflexive questions on our graphic abortion projects.

Our own collaborative abortion graphic novels emerged from our recognition of the power and potential of telling abortion stories. Given our research experience in abortion in Latin America (CF) and India (RN) we wanted to centre narratives that are less represented, heard in the global North given the predominance of the US in graphic reproduction scholarship and storytelling about abortion storytelling (Belfrage, Didier & Vázquez-Quesada 2021). It was also important for us to show complex, accurate abortion experiences that go beyond typical ‘sad and bad’ representations. We therefore heeded Baird and Millar’s (2019: 1122) call to ‘search out and amplify representations that are positive and that centre the multi-dimensional experiences of subjects beyond the white middle-class woman with the resources to choose’, refusing to further narratives that perpetuated abortion stigma. Finally, we wanted to produce abortion graphic narratives that rejected individualistic, neoliberal representations of ‘choice’ that too often abstract experiences from the structural and collective. This echoes Carly Thomsen’s (2013: 155, emphasis in original) hope that ‘we can begin to imagine *collectively* how our discourses and approaches would necessarily shift if we moved to view abortion as something *worthy of celebration*’.

Out of these goals came Cordelia’s collaboration with the Mexican organisation Fondo MARIA, *Será Deseada*, a graphic novel about three women’s experiences accessing abortion in Mexico City and Rishita’s collaboration with the India-based Asia Safe Abortion Partnership (ASAP), producing *Nirnay*, a graphic novel tracing six women’s abortion experiences in rural India.

### Reflexive Abortion Storytelling

#### Situated Acts

**CF: Rishita, what made you want to create a graphic novel as part of your work?**

**RN:** In 2017, I was collecting data on women’s abortion trajectories in India as part of my PhD. I spent a lot of time in primary health centres (the frontline of the Indian public health system, particularly in rural areas) and I was struck by the number of graphic images and posters warning against sex-selection and sex determination, but I never spotted one that discussed abortion itself. And here, I mean ‘graphic’ as both a visual *and* that it was explicit, using rather violent imagery: crushed pigeons or images of (feminised) babies or foetuses.

Abortion in India has been legal under broad grounds since 1971, but many - including almost all of my respondents-do not know what the law is. Abortion is often conflated with restrictions around sex determination (which covers and regulates diagnostic techniques and machinery like ultrasounds rather than abortion itself). Anti-sex selection and sex determination features in a number of public health campaigns and messaging, but abortion does not have a similar mechanism. To me, this links to a broader reluctance or taboo around abortion, even though it is such a common experience. I started toying with the idea of some kind of graphic output - a poster, a comic, a ‘zine - when I was analysing my data and writing up my PhD. It felt like it would be one way of translating my data into something more than just a bunch of academic papers. I did not manage to incorporate it into my PhD, but I decided to draw on this for a postdoctoral grant application for 2020-2021.

Instead of thinking about abortion visuals more broadly, I wanted to draw on my PhD data to co-create graphic novels with frontline health workers in India - a cadre of key community health intermediaries called Accredited Social Health Activists (ASHAs). I intended to draw on women’s narratives of abortion care-seeking and work with ASHAs to identify different points along the trajectories that they were involved or could have been, highlighting not just how they could have supported access but also perhaps hindered it. Just as I submitted my application to the Economic and Social Research Council (ESRC) in March 2020, we were hit by the first wave of the pandemic.

In June 2020, I learnt that my application was successful, and I was due to begin the project in September 2020. All my initial plans - travelling to India, meetings with ASHAs and ASHA unions, piloting our methodology and running the co-creation workshops-were scuppered. Apart from the travel lockdowns, I was concerned about adding to ASHAs’ burden - as frontline workers, they were now tasked with contact tracing amongst other duties and were carrying an immense workload. I also did not think my project was essential in the midst of a pandemic-many of my friends and colleagues in India did not have the time or the energy to focus on this: there were far more pressing matters like ensuring access to abortion services in the midst of a lockdown or organising food drives. I needed to work out another set of plans that did not cross my ethical boundaries and, for a long time, I considered abandoning this aspect of my postdoctoral grant…

What about you, Cordelia? What sparked *Será Desada?*

**CF:** *Será Deseada* came about when I was putting together a grant application to do a research project that would break the cycle I was in at the time of fixed-term teaching contracts. I applied for an Economic and Social Research Council (ESRC) New Investigator Grant and part of their requirement is to put together a ‘pathway to impact’ plan. I immediately knew that a non-academic ‘output’ that I wanted to co-produce was a graphic novel after reading Gemma Sou’s *After Maria* (2019) and teaching using the work of PostiveNegatives, a group who create graphic narratives about social issues based on ethnographic research. This then led me to research existing work about abortion in Latin America and I was inspired by the abortion narratives published by abortion activists and accompaniment groups such as the fanzine *Abortos en Plural* published by Las Comadres in Ecuador (2020) and Aborto Aquí Te Cuento (2018), a collection of abortion experiences written in Spanish and Náhuatl by Red Necesito Abortar in Mexico. I had previously met Sofía Garduño Huerta who works for Fondo MARIA, a Mexico City based organisation who support women who want to end their pregnancies and change the discourse in Mexico to highlight the positive consequences of abortion. When I received the ESRC New Investigator Grant in 2020 I contacted Sofía who liked the idea of a graphic novel and we began conceptualising it along with my colleague at the time, Sandra Rodríguez.

**Fig. 1:**
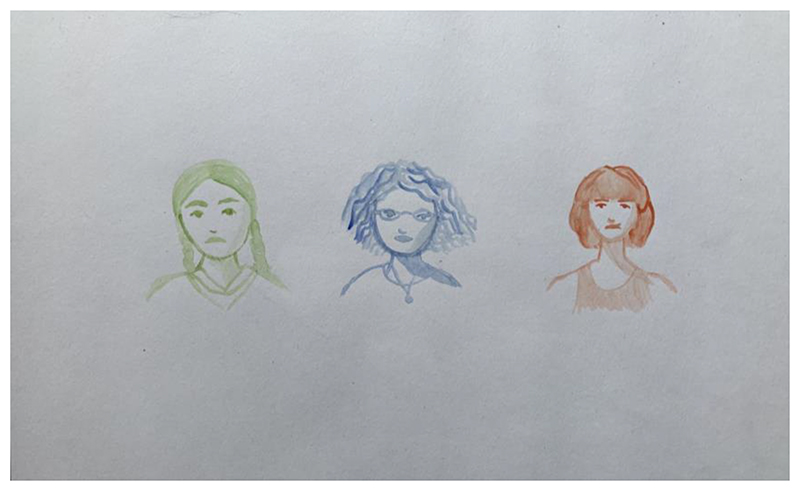
Sketches by Sandra Rodríguez as part of the pitching process

This planning stage coincided with the Covid-19 pandemic and so I was unable to travel from where I was based in the UK to Mexico City and it also meant changing the nature of the graphic novel. I had initially planned to hold a workshop with people who had had abortions in Mexico to storyboard ideas and have them lead the narrative and aims. As we could not arrange anything in person and running this type of workshop online did not feel quite right, we adapted the process. Instead, Sandra and I developed a structure and narrative outline with some rough sketches which we pitched to Fondo MARIA (fig 1). This became a refinement process where they drew on their experiences and expertise and shared ideas with more people at the organisation. We then came to a set of three stories that we felt captured a range of abortion experiences in Mexico both before and after the legalisation of abortions in Mexico City. We then hired a scriptwriter, Gabriela Jáuregui, and an illustrator, Eréndira Derbez, to finalise and create the graphic novel. As a team we all gave feedback and approved drafts and the final version of *Será Deseada* was published and launched at an online event on 28 September 2021, International Safe Abortion day.

How was the process for you, were there any challenges you encountered along the way?

**RN:** Quite similar to you, the pandemic meant I had to adjust a lot of my plans too. My biggest challenge was whether or not it was possible to produce the graphic novel under (remote) pandemic conditions - and whether it would be ethical to even attempt to produce a variation of my original plan. By December 2020, given pandemic conditions in the UK and India, I shelved the idea entirely. I still kept talking about it though, as some kind of unrealisable dream!

And then in March 2021, fortuitously, I was invited to present on an abortion panel with colleagues from India. I reconnected with Dr Suchitra Dalvie, a gynaecologist and staunch abortion advocate who leads the Asia Safe Abortion Partnership (ASAP) based in Mumbai, India. Suchitra and I know each other from my previous life working with abortion and reproductive rights groups and collectives, and it felt a little bit like I had come full circle. I reached out to Suchitra about the project and to see if there was any capacity/interest in working together on this.

Luckily for me, the ASAP team- Dr Suchitra Dalvie, Nandini Mazumder, and Ayesha Bashir-were enthusiastic about the project and brimming over with ideas and we began collaborating in May 2021. We agreed to utilise narratives from the qualitative interviews I had conducted, and on some of the key themes we wanted to tackle. This was all relatively smooth-we had similar visions and ideas for the project and its purpose. Like you, though, I needed to negotiate a lot of what kind of work was possible, particularly remotely!

What continued to be difficult was that we had to keep making space for the pandemic, whether in how we worked (across time zones, over Zoom) or in how we understood the devastating impact of the second wave of the pandemic in India for the team, our communities, and for the project. We had covid infections, long recovery periods, internet troubles, care responsibilities, and competing priorities (including me trying to find a new job for September 2021!). We’re still finishing up the graphic novel (in June 2022) with a wonderful Indian artist-Shourya Dubey- and it continues to be a slow, steady collective labour of love and vision.

The stop-start trajectory of the project was hard to manage within the UK university system, not just because it was difficult to constantly explain how much the second wave had affected the project but to grapple with what these changes (remote, no workshops or travel) meant budget wise. I had to detail these for the funder when requesting a no-cost extension (which was eventually granted) and the bureaucracies of the system - making sure the wording is correct, the constant justification-it all just highlighted how unbending these systems can be, even in the midst of a global pandemic. I really struggled with this aspect of producing work (slowly).

Was there a challenge you hadn’t anticipated or weren’t quite prepared for?

**CF:** I can absolutely relate to those bureaucratic challenges. I hadn’t anticipated how complicated it can be to negotiate between UK university procedural systems and a non-academic organisation in Mexico. For example, UKRI, the non-departmental public body of the UK Government that funds research, stipulates that recipients of funding through UKRI grants must complete a due diligence questionnaire. This is an extensive document that requires organisations to provide documentation on a range of points such as their anti-fraud policies, their recruitment policies, and their financial audits. This same document is sent to multinational corporations and volunteer-run groups in the global South. In my experience, in practice, the university research management team is understanding that not all organisations will have clear documentation for every single question, but this is an intimidating and off-putting document for organisations to receive. In addition, organisations are sent and asked to sign a substantial consultancy agreement that sets out the terms regarding payment, intellectual property, indemnity insurance, and so on. Again, this same agreement is used regardless of the size of the organisation and again it is an intimidating and stressful document. There are additional barriers such as the lack of a legal expert to understand the complex legal terminology and this is exacerbated when the collaborators’ first language isn’t English. This was my first experience of these kinds of contracts and I was left feeling a sense of distrust of both my collaborators and of me as an academic able to be relied upon to collaborate with appropriate organisations. While I understood that these levels of paperwork exist to prevent fraud and theft, when it is only a matter of a few thousand pounds, the level of bureaucracy can feel disproportionate.

**RN**: Given these asymmetries of power, how do you work collaboratively - especially remotely - with groups in the Global South? How do you contend with power, or with any tensions that arise?

**CF:** There were multiple axes of difference that were present throughout the project. For example, with me being a white British researcher I was coming from a context of difference that was exacerbated by me being ‘in charge’ of the funding and legal documents. This was a strange negotiation for me as I felt more ‘on the side’ of the collaborators I was working with but it would be naive to ignore the power involved in being the one who determines the terms of service and signs off the work for payment. All of the paperwork obstacles that I mentioned earlier reinforce this power dynamic and it makes it hard to feel like an even and equal collaboration.

The question then of whether it is possible to do collaborative work with a feminist ethic of care is one that that i am still thinking through. Where possible I tried to step back in the key decisions about representation and made sure that everyone we collaborated with was rooted in the Mexican context. I’m working on two other collaborative projects now and am trying to take what I have learnt from the *Será Deseada* project into those. In particular, I’m learning to see all the paperwork as a necessary bureaucratic stage we need to get through and after that it’s just me and my collaborators but contending with being located in a British university is always going to make these questions of power complex. What have your experiences been?

**RN:** Rather similar concerns to yours around reciprocity and a feminist ethics of care! I too was very conscious of holding power across different domains - institutionally, funding, the data. I tried to contend with power by being as explicit as I could be - with myself and the team - about how, when, and where we might confront it. One of the ways I attempted to grapple with this imbalance was by being transparent about the budget, and collectively allocating budget lines for different activities (that we also decided on together). Here, I was led almost entirely by the ASAP team. From the outset, I wanted to make sure that anything we produced was open access and could be (on request) shared with anyone who wanted to utilise the raw materials. This was included in my original postdoctoral application, so I could leverage this to ensure that the contract with ASAP gave them equal rights over the materials. In this same vein, we are planning to write a number of co-authored posts about our work and approaches – another aspect of knowledge building that is valuable to me.

#### Representations

**CF: Rishita, what conversations did you have about how to write feminist abortion narratives and attempt to show ‘no bad abortions’?**

**RN**: Many of the discussions specific to our writing and depictions for the graphic novels came out of our conversations around reproductive justice and political analyses of abortion and reproduction in India. It sometimes felt as though we discussed everything but abortion itself! I think, however, this was essential to how we understood abortion - as a facet of women’s everyday lives, as a common occurrence, as shaped by larger political, social, economic forces…

**Fig. 2:**
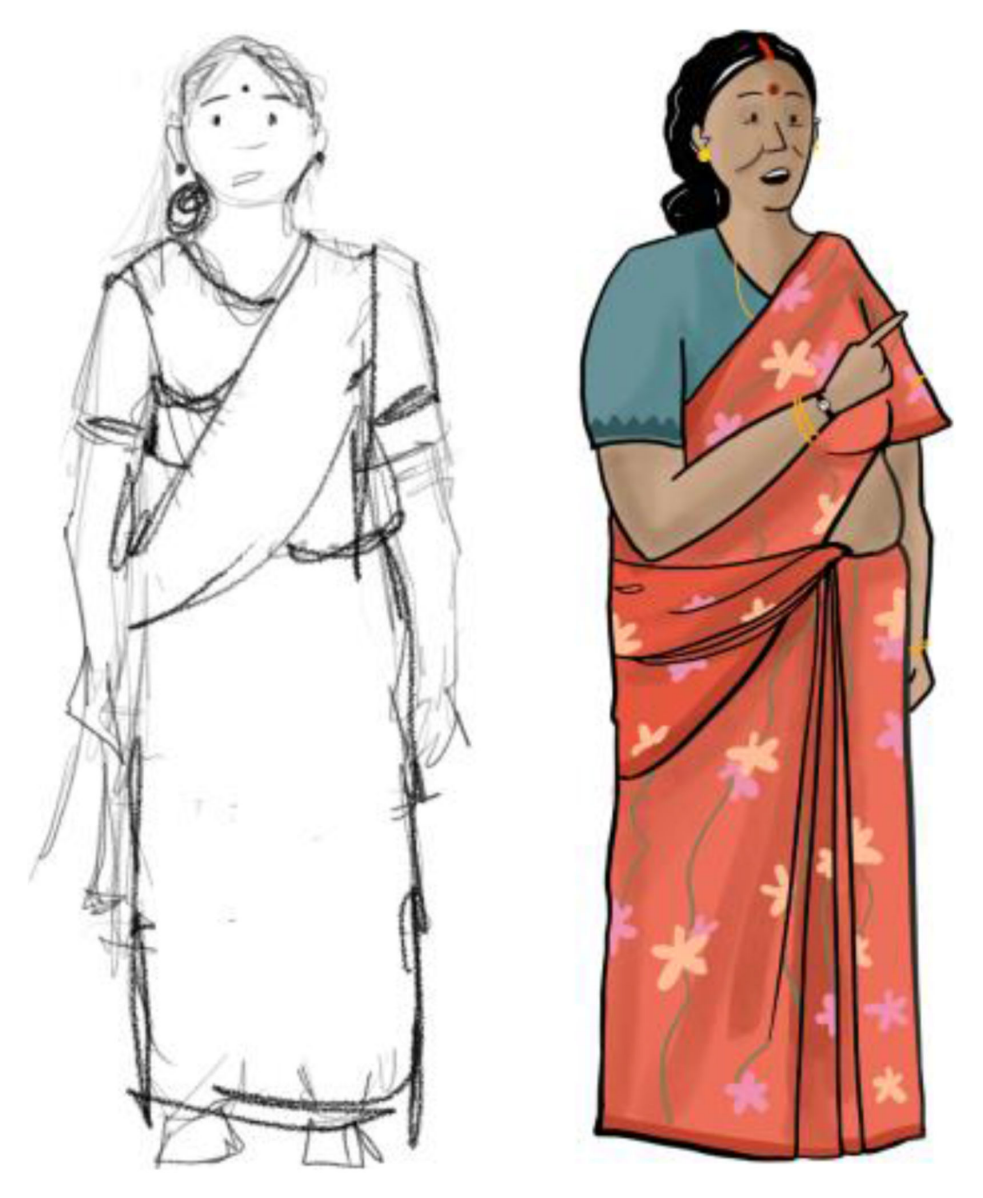
We wanted to showcase the range of actors involved in shaping abortion trajectories in *Nirnay*. This is Shourya’s sketch of an ‘aunty’ figure in one of the narratives.

Since we were drawing on narratives from the qualitative interviews, we focused on *whose* narratives we were drawing on and whether we had a range of backgrounds (e.g., ages, marital status, reasons, caste, class) and experiences (e.g., varying levels of supportability (Macleod 2016), emotions), *which* abortion methods (e.g., self-managed, medical, surgical), and the kinds of actors involved in the trajectory (Fig 2.). We were also very keen to engage with abortion-seekers’ range of emotions and depict uncertainty, ambiguity, relief, resolve, and sorrow equally.

We explicitly discussed intersectionality and ensuring the six narratives did not offer singular or stigmatising ideas of abortion seekers or abortion experiences. Our discussions on the social and structurally violent conditions for Dalit and Muslim communities in India underpinned this. We were determined to ensure that abortion-seekers’ agencies and tenacity were clear - that even when encountering judgement, refusal, or disempowering conditions, their autonomy and efforts to circumvent or navigate these barriers were evident. We really did not want to depict ‘passive victims’ but instead underscore how structural conditions shaped and influenced trajectories and autonomies. We decided to work with first person narratives to help achieve that and avoid the simplistic binary or oversimplification that Ludlow (2020b) warns against.

Based on the data and our discussions, I drafted six narratives for discussion. To avoid any risk of triangulation and secure anonymity further, each narrative is a composite (like your approach!). I categorised data by similarity (age, reason, trajectories) and layered experiences – for example, heightening interactions with clinicians or adding to objections voiced by partners or family members. While drawing on existing data roots the depictions in everyday realities, it is also constrained by the narratives captured in the study. Except for one respondent (who had a very specific set of circumstances that I was wary about including), all the other respondents in my study were currently married. We’ve been mulling over how to incorporate or nod to experiences of those who are not currently married, and their trajectories to care.

Coming back to the commitment to centre agency and autonomy, we flirted with the idea of keeping the researcher (i.e., me) as a mechanism to link the narratives. However, we felt this would distract from the narratives. I also worried it would shift the audience for and purpose of the comic, pulling it towards an academic exercise where I would be obliged to depict my insider-outsiderness and grapple with reflexivity as an Indian academic in the UK. We decided to rely on linking the stories through overlaps between characters in the background, or in locations. We also agreed to include an introduction, offering an overview of the context and politics of abortion and the laws in India. This serves three purposes: introducing and contextualising the project, an information sharing and educating function, and like other feminist abortion comics, it explicitly challenges the idea of abortion as shameful or taboo (McGovern and Eve 2019).

What about you, how did you and the team come to infuse *Será Deseada* with a feminist and reproductive justice approach?

**Fig. 3:**
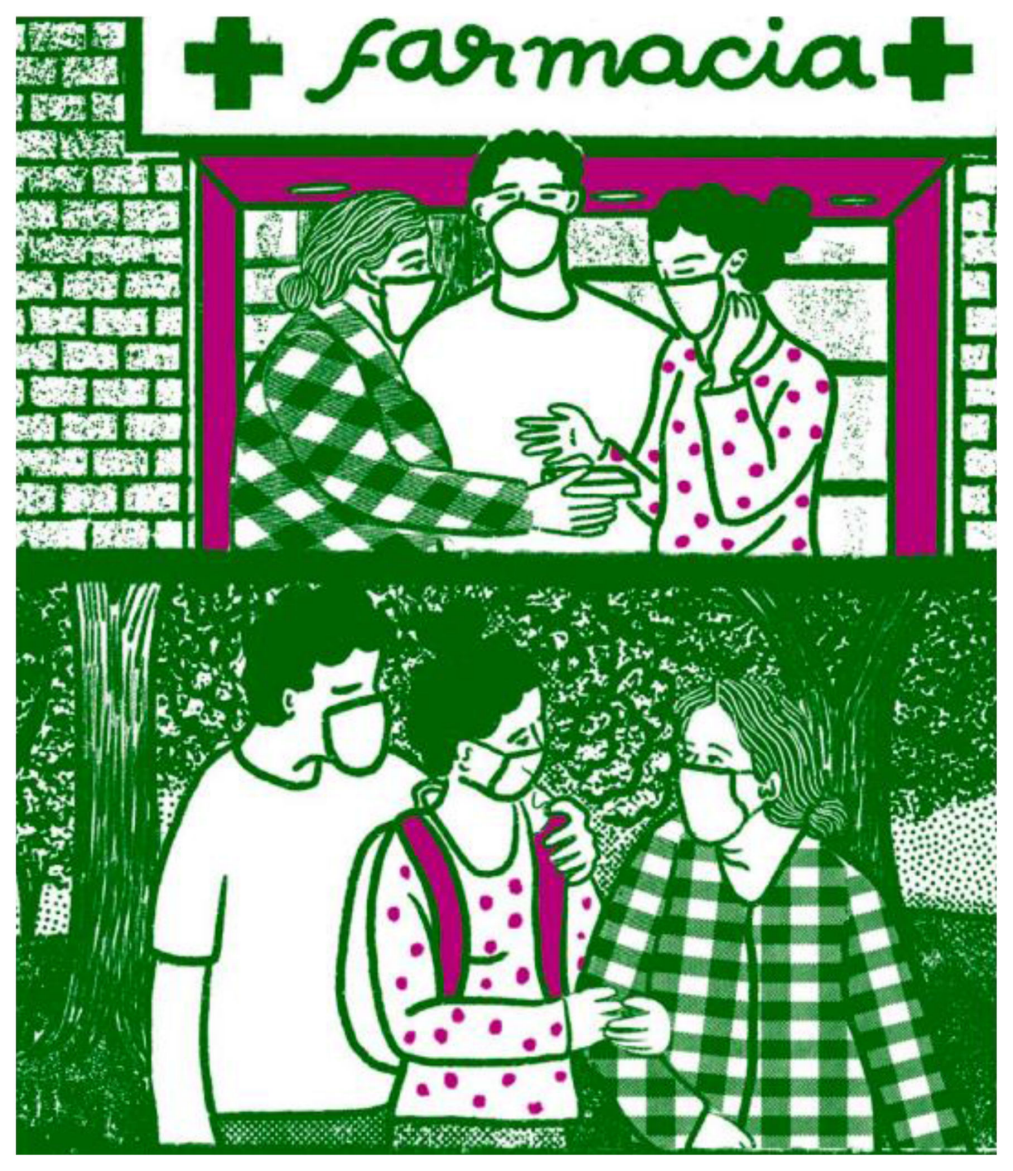
In Será Deseada, we showed the characters with their families to emphasize that abortion is part of ordinary life and to show the importance of the collective. Here, one of the characters is shown with her partner and grandmother.

**CF:** With *Será Deseada*, we had conversations throughout the process about how to represent abortion, around what stories we wanted to emphasize, ‘who’ the characters should be, and what their abortions should look like. The aim of *Será Deseada* was to provide realistic depictions of accessing an abortion in Mexico and show how access has changed over time. Our collaborators at Fondo MARIA had a wealth of experience speaking to people about their abortions and supporting them to have safe abortions, which meant that they were able to draw on real experiences to create composite storylines that did not compromise any individual’s anonymity. It was important to us as a team that the abortion wasn’t the sole thing we learnt about the characters and so they were shown in their homes, with their families, and in ordinary situations (fig. 3). We also wanted to highlight the collective nature of abortion and so throughout the novel the reader learns that the three stories are interconnected. Like with yours we also decided to have a ‘prologue’ so Sandra and I wrote a piece that explained the legal context and our approach in order to make it explicitly political.

There were some discussions about the implications of what we wanted to show that highlighted the limits of my knowledge as a British academic and reinforced the necessity of collaborating with activists in my research site. For example, I suggested early on that one of the three characters should be queer or bisexual to refute assumptions that only straight women have abortions. However, Fondo MARIA made the important point that with the climate around abortion, sexuality, and ‘anti-genderism’ in Mexico, anti-abortion critics look for reasons to denigrate those seeking abortions (even fictional characters). Therefore, queerness could be used to say ‘look, it’s sick people who have abortions’. As Belfrage, Didier & Vázquez-Quesada (2021) have noted, using personal stories about abortion in contexts where abortion is restricted and stigmatized is challenging when those stories can reinforce stigma or be co-opted by anti-abortion groups. We therefore made the decision not to have an openly queer character. We had these kinds if discussions about so many aspects of the graphic novel- from the role of religion, to whether we wanted to show the potential of abortions with plants, to what the relationship status of the characters should be, and so many other things. With all aspects it was important for us to uphold the absolute right to bodily autonomy and show the love, solidarity, and resistance that underpins abortion access in Mexico.

What about design, how did you decide how to approach your graphic novel aesthetically?

**RN:** This was a really fun part – we all came to a Zoom call with images or links to different art/styles we liked and talked it through. This was everything from specific motifs and patterns (e.g., references to Indian textile motifs) to art styles (e.g., Warli or Madhubani) or artists (e.g., Jamini Roy). We were also unanimous in wanting bright colours in our comics– we were all a bit tired of dark palettes when depicting abortion! This was something we considered when finalising the artist that we wanted to work with. Shourya’s work was especially striking for the colours she used and her attention to detail–her backgrounds, whether depicting the centre of the village or someone’s front room, are rich and textured.

For every narrative that Shourya produced, we offered collective feedback. One of my favourite discussions was around the depiction of blood over the course of a self-managed abortion. We all agreed we wanted to show this– not to shock, but to normalise. We ended up spending a fair bit of time considering what shade of red we ought to use! I suppose this speaks, in some way, to Ludlow’s (2019) critique of the absence of the aborted foetal body in pro-choice narratives.

**Fig. 4:**
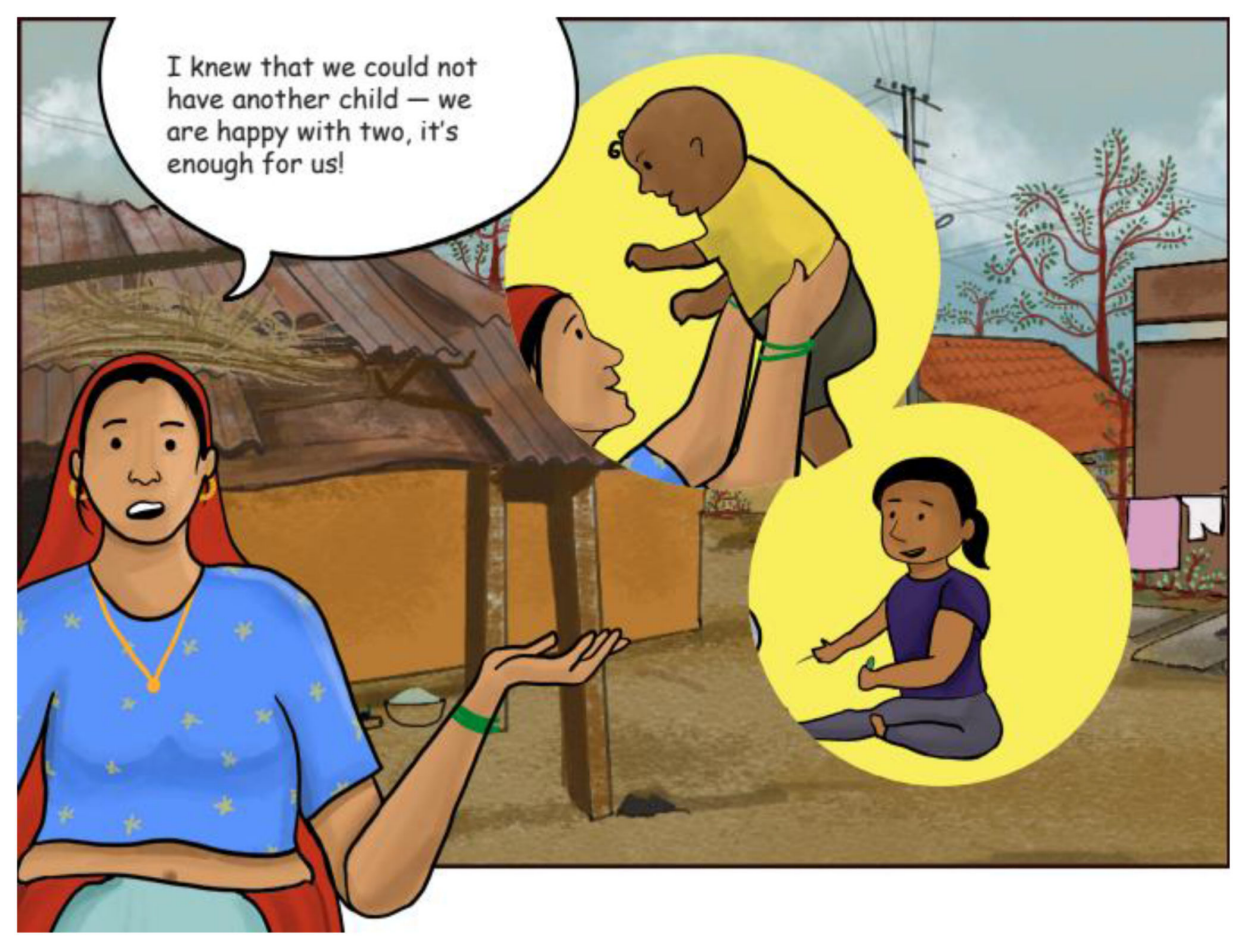
An extract from *Nirnay* to highlight the complexity of peoples’ lives.

Ayesha, from the ASAP team, and I added additional depth and explanations to aid Shourya in interpreting which elements to highlight (e.g., the need for secrecy or the sense of urgency) or include (e.g., children playing in the background) to realise our visions of abortion narratives that captured the complexity of peoples’ lives and abortion trajectories (Fig. 4).

I love the palette and visualisation in *Será Deseada*! How did this come about in your project?

**Fig. 5:**
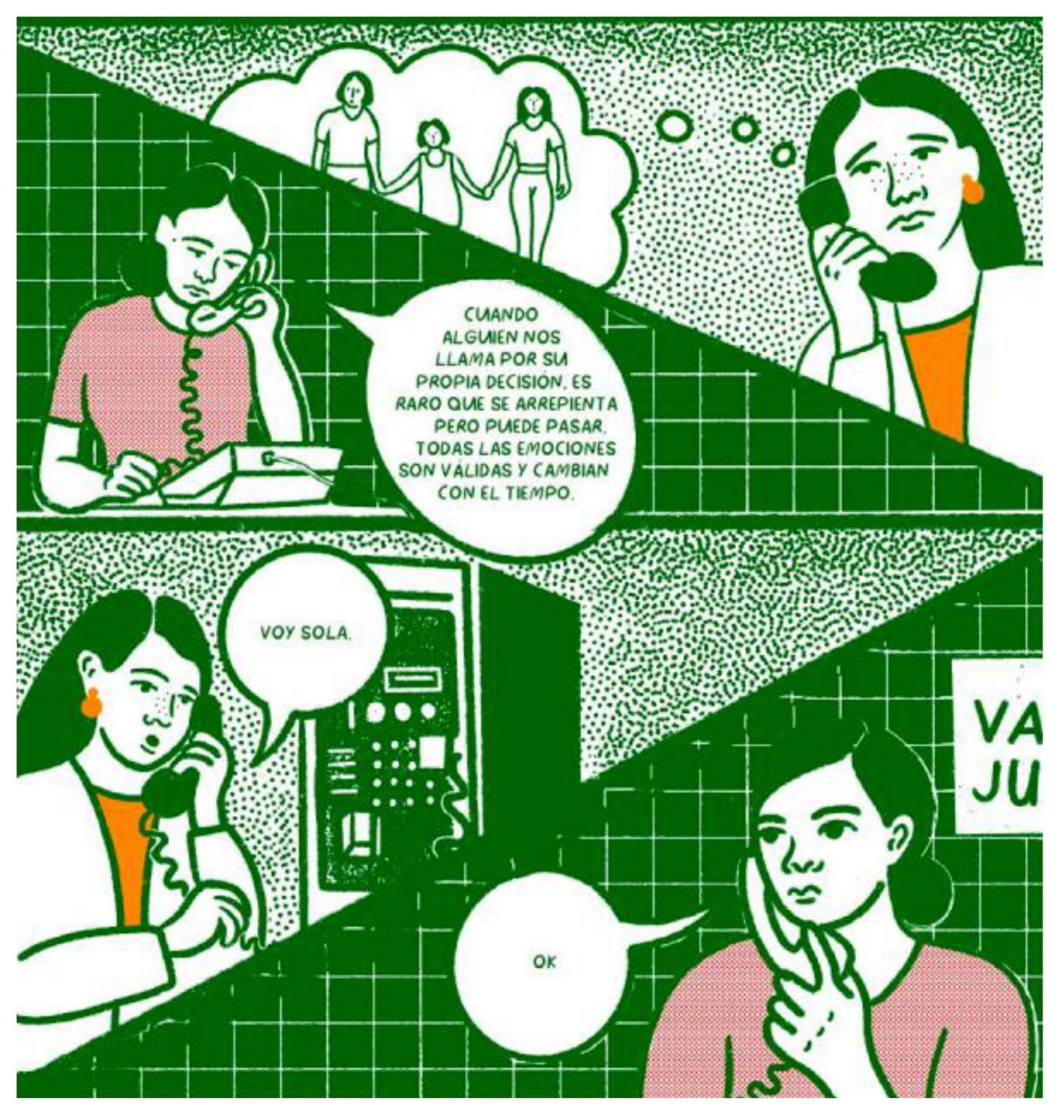
An extract from Será Deseada to show the use of colour

**CF:** We had fewer explicit discussions about aesthetics as we tended to take more about content and political representation. The illustrator, Eréndira Derbez, was given artistic licence and checked in with us as a team to see what we thought. The resulting style balances simplicity and expression and the predominant colour of the graphic novel is green, the colour of Latin America’s *Marea Verde* [Green Tide]. We spoke a lot about how we would portray three stories as we knew we didn’t want them separate and so each of the three characters has her own colour so that the reader can differentiate between them as the stories interweave (fig. 5).

**RN:** Cordelia, is there anything you wish you had done or regret not being able to do in this project?

**CF:** I think when you are so immersed in the area of research you know so many complexities and nuances that you want to be able to include every possible type of experience, but doing that would create a chaotic and unclear narrative. We therefore went for ‘simple not simplistic’ which makes for a more convincing graphic narrative but does mean we were not able to show abortion experiences outside of Mexico City or particular barriers that queer people face as I mentioned earlier, for example. I do also wish that we had had the opportunity to run that workshop to develop ideas with people who have had abortions themselves as I think that would have led to some fascinating insights and could have been quite a powerful thing for people to have been a part of, I think, given that sharing abortion stories can be empowering in the right circumstances (Woodruff et al. 2020; Belfrage, Ortiz Ramírez and Sorhaindo 2020). Is there anything you wish you had been able to do but couldn’t?

**RN:** I hear you - I wish we’d been able to run workshops as well! I also really like the idea of ‘simple not simplistic’ too - I hope that’s what we’ve managed as well.

One thing I really wanted to do was include experiences of trans, non-binary, and gender non-conforming persons in these depictions. We did briefly discuss adding a fictionalised narrative, but as we were limited to drawing on data from the existing project; we decided against it. This is something I would really like to redress in the future–not just by ensuring that the project works closely with LGBTQI+ communities but also that these narratives draw on their experiences and data! Something else we discussed was whether or not to include the pandemic and the imposed barriers– lockdowns, quarantines, burden of testing etc. Again, given that these trajectories were deeply affected by the pandemic and we did not have the data to draw on, we parked that for ‘next time’.

### Conclusion: A Framework for Producing Graphic Abortion Narratives

Our reflections on the creation of graphic abortion narratives contribute to the burgenoning graphic abortion and graphic reproduction literature. We understand our work as a techné of abortion (Faris, 2019), challenging abortion stigma and contributing to a shift in conceptualisations of abortion.

We identify two main approaches to graphic narratives in academic practice - (i) the ethno-graphic novel (Dix and Kaur 2019) and (ii) retrospective (re)presentation (Rumsby, 2020). In detailing our collaborative processes and engaging with a call for feminist reflexivity (Belfrage, Didier & Vazquez-Quesada 2021), we build on Dix and Kaur (2019) and Rumsby (2020) to offer an adapted framework for co-producing graphic novels. This framework, while immediately relevant and linked to feminist approaches and to depictions of abortion and reproduction, can be applied to other topics and disciplinary approaches as well.

Dix and Kaur (2019) urge shifting away from using drawings and visuals for ethnographic field notes alone to a more representational practice to ‘create synchronous affective intensities’ (p. 76). The ‘ethno-graphic novel’ draws on ethnographic fieldwork and collaborative approaches that include research participant feedback on visuals and sketches. Participant contributions in the ethno-graphic novel may be edited or embellished, but straddle a line of truth/testimony and fiction. Dix and Kaur (2019) propose a theory and practice of ‘vérités graphiques’ (literally, graphic realities) which enables an interrogation of this blurring of fiction and truth and challenges the ‘presumed objectivity of what is seen, experienced, co-created, and revealed’ (90). The ethno-graphic novel enables the retelling of experiences through individual testimonials and graphic representations, while simultaneously allowing for reflections of ‘truth’ in these fictionalised representations (p. 107).

Rumsby (2020), building on Dix and Kaur (2019), offers a method of ‘retrospective (re)presentation’ which entails ‘using the visual to offer alternative modes of (re)presentation to the written ethnographic text’ (p.7). Rumsby (2020) details their project of retrospectively creating an ethno-graphic novel, where the initial ethnography did not utilise visual notes or recordings. The focus here is on the collaboration between the researcher and the illustrator, rather than that between the researcher and participants (as in Dix and Kaur 2019). They detail how this process highlights the author’s voice, directs interpretation and structures the narrative (ibid: 18), underscoring drawing as a (re)presentational practice.

In our graphic narratives, we offer a third approach: vérités graphiques co-produced with activist groups (i.e., Cordelia’s work with FONDO Maria and Rishita’s project with the Asia Safe Abortion Partnership). Similar to Dix and Kaur (2019) and Rumsby (2020), our projects draw on existing testimonies and experiences, hold collaboration central to the production of the graphic narratives, and tread the truth/testimony and fiction line. Where we depart from their approach is in the nature of the collaboration and co-creation, and the kinds of graphic representations it enables.

*Será Deseada* draws on the experiences and intimate knowledge(s) of feminist activists who are keenly aware of the range and texture of abortion experiences, the confrontations, and tensions. In drawing on real life experiences, these narratives offer a plurality of experiences to ‘create synchronous affective intensities’ (Dix and Kaur 2019: 76) that are tied to the feminist impetus for transformation. In turn, these narratives call on readers to engage with the range of experiences not just as observers, but participants in the larger call for change. Similarly, Rishita’s work on *Nirnay* centred around collaborating with a feminist activist group. *Nirnay*, however, melds this with Dix and Kaur’s (2019) ethno-graphic approach by drawing on qualitative data to create composite narratives.

Like Rumsby (2020), we both worked closely with the illustrators, who had creative agency. The illustrators, however, were part of the larger activist teams and spaces; already familiar with the contexts, the issues, and the feminist approaches. Their knowledge and ‘insider’ status adds another layer of (re)presentation to the text and the ‘truth-fiction spectrum’ (Dix and Kaur 2019). Enabling a collective approach, this effort attempts to enact feminist reflexivity as a situated act (Belfrage, Didier & Vázquez-Quesada 2021; Nencel 2013), and heed the impetus to consider *whose* voices and knowledge are depicted, valued, and amplified.

As more social science disciplines see a ‘graphic narrative turn’ (Dix and Kaur 2019), especially in the context of increased attention on ‘research impact’ and ‘knowledge exchange’, we offer our framework as an additional method or way of thinking about the production of graphic or visual narratives. While focused on abortion in our iteration, we believe the feminist ethic and approach translates across disciplinary or topic boundaries. The explicit attention to reflexivity, collaborating with local activists, and infusing a collective approach to (re)presentations, can be adapted for most projects. For instance, we can imagine this approach being adopted by those working on topics as broad ranging as the climate emergency, health issues, labour rights, migration, and LGBTQI+ activism.

Our modified collaborative, situated, and collective approach makes explicit the feminist demand for change– not just as a form of witnessing but in transforming the reader/observer’s role to one of a participant. This feminist ethic enables a techné of abortion (Faris 2019), educating the reader/observer about abortion and challenging stigma. It sits within a broader feminist praxis of self-help through educating, and enabling information-sharing. Through this, it destabilises static and reductionist views of abortion; creates and holds space for complexity and ambiguity (Ludlow 2020b); and offers new imaginaries and discourses of abortion.
